# Submicron particle docetaxel intratumoral injection in combination with anti-mCTLA-4 into 4T1-Luc orthotopic implants reduces primary tumor and metastatic pulmonary lesions

**DOI:** 10.1007/s12032-021-01555-1

**Published:** 2021-07-31

**Authors:** Holly Maulhardt, Alyson Marin, Holly Hesseltine, Gere diZerega

**Affiliations:** 1 US Biotest, Inc., 231 Bonetti Drive, Suite 240, San Luis Obispo, CA USA; 2 NanOlogy, LLC., 3909 Hulen Street, Fort Worth, TX USA

**Keywords:** Docetaxel, NanoDoce, Breast cancer, Immunotherapy, Anti-mCTLA-4, LSAM-DTX

## Abstract

We describe here characterization of the response of local and metastatic disease and immunomodulation following intratumoral (IT) injection of submicron particle docetaxel (SPD) administered alone or in combination with systemic antibody anti-mCTLA-4 (anti-mCTLA-4) in the metastatic 4T1-Luc2-1A4 (4T1) murine breast cancer model. In-life assessments of treatment tolerance, tumor volume (TV), and metastasis were performed (*n* = 10 animals/group). At study end, immune cell populations in tumor-site tissues and peripheral blood were analyzed using flow cytometry. Signs of distress typical of this aggressive tumor model occurred across all animals except for the combination treated which were asymptomatic and gained weight. TV at study end was significantly reduced in the combination group versus untreated [43% reduced (*p* < 0.05)] and vehicle controls [54% reduced (*p* < 0.0001)]. No evidence of thoracic metastasis was found in 40% of combination group animals and thoracic bioluminescence imaging (BLI) was reduced vs. untreated controls (*p* < 0.01). Significant elevations (*p* < 0.05) in CD4 + T, CD4 + helper T, Treg, and NKT cells were found in tumor and blood in SPD or combination treatment compared to controls or anti-mCTLA-4. Combination treatment increased tumor-associated CD8 + T cells (*p* < 0.01), peripheral B cells (*p* < 0.01), and tumor associated and circulating dendritic cells (DC) (*p* < 0.05). Tumor-associated NK cells were significantly increased in SPD ± anti-mCTLA-4 treatments (*p* < 0.01). Myeloid-derived suppressor cells (MDSC) were reduced in bloods in SPD ± anti-mCTLA-4 groups (*p* < 0.05). These data demonstrate that both SPD and anti-mCTLA-4 produce local anti-tumor effects as well as reductions in metastasis which are significantly enhanced when administered in combination.

**Supplementary Information:**

The online version contains supplementary material available at 10.1007/s12032-021-01555-1.

## Introduction

We developed a submicron particle production technology that produces particles composed of approximately two billion docetaxel molecules with a mean particle size of ∼ 900 nm and a specific surface area (SSA) of 19.67 m^2^/g or 2.1x greater surface/weight ratio than docetaxel drug substance [1]. SSA is a measure of the particle surface area-to-mass ratio and is directly proportional to the rate of drug release from the particles. The resulting drug, SPD (NanoDoce®; also described as large surface area microparticle docetaxel or LSAM-DTX), is under evaluation in a clinical trial of high-risk non-muscle invasive and muscle invasive bladder cancer by injection into the tumor resection site followed by induction and maintenance cycles of intravesical instillations (Clinicaltrials.gov identifier NCT03636256) [2]. Additional clinical trials using local administration of SPP (NanoPac®) were completed in recurrent ovarian cancer [3, 4], focal prostate cancer (Clinicaltrials.gov identifier NCT03077659), and pancreatic mucinous cysts (Clinicaltrials.gov identifier NCT03188991) [5] and are ongoing in IT injection of lung cancer (Clinicaltrials.gov identifier NCT04314895), focal prostate cancer (Clinicaltrials.gov identifier NCT04221828), and locally advanced pancreatic cancer (Clinicaltrials.gov identifier NCT3077685).

Previously, we reported that IT injection of SPD into uro-oncologic xenografts (786-O, clear cell renal carcinoma; UM-UC-3, transitional cell bladder carcinoma; and PC-3, prostate carcinoma) resulted in reduction or elimination of tumors more effectively than IV docetaxel [1]. High levels of docetaxel were retained in SPD-injected sites up to 50 days post-treatment. We also reported that injection of SPD into syngeneic murine xenografts models of renal adenocarcinoma (Renca) significantly reduced or eliminated primary tumor growth compared to IV docetaxel and affected rates of tumor growth in secondary tumor implants [6]. In addition, CD4 +, CD8 +, and Treg populations were increased in peripheral blood from animals following SPD treatment. Data from preclinical and clinical studies demonstrated that local administration of SPD or submicron particle paclitaxel (SPP) provided increased anti-tumor activity directly at the primary tumor site [1, 6–9] and indirectly through enhanced immune effector cell response in peripheral blood [1, 5, 6]. Further benefit is seen following submicron particle taxane IT therapy with reduced systemic toxicity compared to IV taxanes therapy [1, 3, 5, 6]. The cytotoxic T lymphocyte antigen 4 (CTLA-4) is a critical receptor that regulates T cell activation and tolerance. CTLA-4 has been shown to inhibit T cell responses and its blockade with the anti-CTLA-4 antibody leads to cell-mediated tumoricidal immunity [10, 11].

In the current study, the local response of 4T1 breast cancer tumors, in which a luciferase gene is fused to tumor cells allowing the extent of thoracic metastasis to be quantified in-life with BLI, were evaluated following IT SPD, intraperitoneal (IP) anti-mCTLA-4, or the combination and compared to IT vehicle and untreated controls. 4T1 is a poorly immunogenic, aggressive, and highly metastatic stage IV murine breast cancer derived from a BALB/c spontaneous carcinoma [12] which, when implanted orthotopically, has a tumor volume doubling time of 6 to 7 days. Disease progression accompanied by reduced body weight gain is a characteristic of the 4T1 model. CD11b + myeloid cells are the predominant infiltrating cells in 4T1 tumors [13] and are primarily granulocytic myeloid-derived suppressor cells (G-MDSC). In addition, minimal T cell infiltration leads to immunologically “cold” conditions in the 4T1 tumor microenvironment (TME). Previously in the 4T1 model, anti-mCTLA-4 in combination with local radiation has been shown to inhibit lung metastasis and increase survival [14]. In this study, changes in tumor volumes and BLI were evaluated over 24 days, at which point adaptive and innate immune cell populations in the primary tumor and peripheral blood were measured by flow cytometry.

## Materials and methods

### Animals and husbandry

Seven-to-eight-week-old female BALB/cAnNHsd mice (Envigo; Indianapolis, IN) were housed in clean rooms controlled to 70 ± 2°F and 30–70% humidity. Animals were fed irradiated Teklad 2918.15 Rodent Diet and water ad libitum.

Clinical observations were performed at least once daily, and body weights were recorded three times weekly. Animals found in distress or moribund condition were euthanized. Necropsies were performed and potential cause of death was assessed; target organs for toxicity were evaluated and presence/absence of metastases was noted.

### Preparation of submicron particle taxanes

SPD and SPP (NanoDoce® and NanoPac®, respectively; CritiTech, Inc., Lawrence, KS) were developed as described [1, 5, 7, 8] to increase IT drug concentrations and residence time through local delivery. SPD is produced using precipitation with a proprietary compressed anti-solvent technology in which dissolved docetaxel drug substance in solution is sonicated into droplets. The solvent is stripped using supercritical fluid CO_2_ which precipitates submicron particles of pure docetaxel approximately 900 nm in size by number-based particle size distribution. The SSA of particles are determined using the USP compendial test Specific Surface Area < 846 > [15]. SPD or SPP is suspended at time of use in a saline-based solution that maintains particle size and can be delivered directly to the disease site using commonly employed techniques including image-guided fine-needle injection, nebulized inhalation, and intravesical or IP instillation.

### Tumor implantation and treatment

4T1-Luc2-1A4 luciferase-enabled mouse mammary gland tumor cells (PerkinElmer, Waltham, MA) were grown in RPMI 1640 media modified with 1 mM NaPyruvate, 10 mM HEPES, and 2.8 mL 45% glucose and supplemented with 10% non-heat-inactivated fetal bovine serum and 1% penicillin/streptomycin/l-glutamine at 37 °C with 5% CO_2_. When expansion was complete, cells were trypsinized using a 0.25% trypsin–EDTA solution. Following cell detachment, the trypsin was inactivated with complete growth medium and clumps of cells were separated, diluted in a trypan blue solution, and cell viability was determined to be 89% using the LUNA-automated cell counter (Logos Biosystems, Annandale, VA). A total of 5.0 × 10^5^ tumor cells in 50 µL were implanted into the #4 mammary fat pad on Day 0 using a 27-gauge needle. No Matrigel was used in the implantation suspension. Cells were maintained on wet ice during implantation and cell viability following implant was determined to be 94%.

Prior to treatment initiation on Day 10, the mice were sorted into groups based on caliper estimation of tumor burden. Treatment began at a mean tumor burden of 96 mm^3^ (range of group means, 93–98 mm^3^). SPD suspensions (40 mg/mL submicron particle docetaxel, 0.4% polysorbate 80/3.2% ethanol in 0.9% sodium chloride for injection, pH 7.4, purity > 99.0%) and vehicle (excipients same as SPD formula) were dosed IT at a fixed volume of 25 µL as a single bolus dose on Days 10, 14, 18, and 22. Antibody anti-mCTLA-4 (Clone 9D9) (1 mg/mL in Dulbecco's phosphate-buffered saline (DPBS), pH 7.2, purity > 95%, (Bio X Cell, Lebanon, NH)) was dosed at 10 mg/kg IP on Days 10, 13, 17, and 20. The combination group received both SPD and anti-mCTLA-4 on the same schedules as the monotherapies.

### In vivo BLI

In viv﻿o BLI images were acquired on Days 16, 23, and 30. Ten minutes (min) following IP administration of 150 mg/kg D-luciferin (15 mg/ml in saline (Promega, Madison, WI)), three to four mice under 1–2% isoflurane gas anesthesia were imaged in the supine position with the primary tumor shielded. BLI was performed using an IVIS Spectrum In Vivo Imaging System (PerkinElmer, Waltham, MA). Large binning of the CCD chip was used, with exposure time adjusted to obtain minimum several hundred counts per image and to avoid saturation.

Images were analyzed using Living Image 4.3.1 (PerkinElmer). The BLI background signal for this study was 1.50 × 10^5^ photon/second (p/s). The thoracic signal was calculated using fixed volume regions of interest to estimate the tumor burden and total flux was calculated.

### Tumor volume evaluations and analysis

Tumor burden (mm^3^) was estimated three times weekly using digital caliper measurements by the formula for the volume of a prolate ellipsoid assuming unit density as:

$${\text{Tumor burden}} \left( {{\text{mm}}^{3} } \right) = (L \times W^{2} ) \div 2$$

where *L* and *W* are the orthogonal length and width measurements (mm).

### Flow cytometry sampling and methods

On Day 34 mice were euthanized and tumors were dissociated into single cell suspension using gentleMACS mouse tumor dissociation kit (Milteny Biotec; Bergisch Gladbach, Germany). Samples were processed in two batches over four hours. Sample acquisition on the Attune NxT Acoustic Focusing Cytometer (ThermoFisher; Waltham, MA) occurred within 36 h. Antibody Capture Beads (hamster or mouse/rat; ThermoFisher) were used as a compensation matrix to determine voltage and settings. Flow cytometry data analysis was performed using FlowJo software (BD Biosciences; Franklin Lakes, NJ). Percentage and absolute counts of cell types within blood and tumor-site tissues were evaluated using the CompLeukocyte™ (Covance; Ann Arbor, MI) panel with the following endpoints: total cells (viability dye negative/debris excluded), CD45 + , CD3 + (CD45 + CD3 +), CD4 + T cells (CD45 + CD3 + CD4 + CD8-), CD8 + T cells (CD45 + CD3 + CD8 + CD4-), Tregs (CD45 + CD3 + CD4 + CD8-CD25 + FoxP3 +), CD4 + helper T cells (CD45 + CD3 + CD8-CD4 + , FoxP3-), CD69 + CD8 + T cells (CD45 + CD3 + CD8 + CD4-CD69 +), PD1 + CD8 + T cells (CD45 + CD3 + CD8 + CD4-PD1 +), and Ki67 + CD8 + T cells (CD45 + CD3 + CD8 + CD4-Ki67 + ; distribution and median fluorescence intensity (MFI)), NK cells (CD45 + CD3-SSClowCD49b-CD335 +), NKT cells (CD45 + CD3 + SSClowCD49b + CD335 +), B cells (CD45 + CD11b-CD19 + SSCLow), CD11b + (CD45 + CD19-CD11b +), G-MDSC (CD45 + CD19-CD11b + Ly6G +), M-MDSC (CD45 + CD19-CD11b + Ly6C +), Macrophages (CD45 + CD19-CD11b + (exclude MDSC)F4/80 +), M1-TAM (CD45 + CD19-CD11b + (exclude MDSC)F4/80 + MHCII + CD206-), M2-TAM (CD45 + CD19-CD11b + (exclude MDSC)F4/80 + CD206 +), and DC (CD45 + CD19-CD24 + F4/80-CD11c + MHCII +).

### Statistics

Prism 6 (Version 6.07; GraphPad Software) for Windows was employed for calculation of descriptive statistics and graphical presentations and statistical analyses of flow cytometry data. Kruskal–Wallis one-way ANOVA was used for multiple comparisons of Day 10 and Day 34 tumor volumes, ∆TV, and body weights and Day 30 BLI data. In the flow cytometry data, outliers were defined as individual results greater or less than the group mean plus three times the group standard deviation and were removed from data sets prior to analysis and graphing. Kruskal–Wallis one-way ANOVA was performed for each cell type for both absolute counts data and percentage of cell types data. The two-tailed statistical analyses were not adjusted for multiple comparisons and were conducted at *p* = 0.05. Results are reported as non-significant (ns) at *p* > 0.05. Significance is reported as *p < 0.05; **p < 0.01; ***p < 0.001; ****p < 0.0001.

## Results

### Tumor volume

**Fig. 1:**
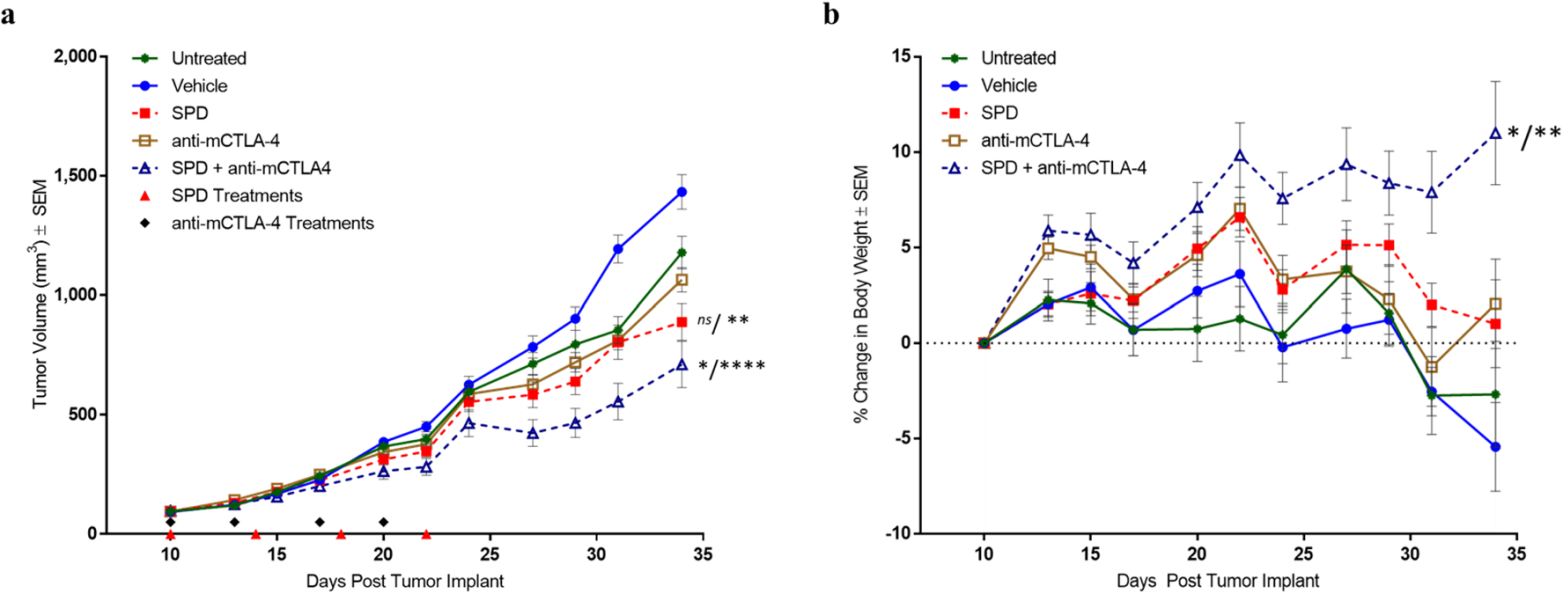
**a** Group mean TV from treatment initiation on Day 10 through end of study on Day 34. *n* = 10 mice/group. All animals survived through Day 34 with exception of one animal in untreated group which exited study on Day 32. SPD suspensions (40 mg/mL) and vehicle were dosed IT on Days 10, 14, 18, and 22 (red triangles). Anti-mCTLA-4 (10 mg/kg) was dosed IP on Days 10, 13, 17, and 20 (black diamonds). The combination group received both SPD and anti-mCTLA-4 on the same schedules as the monotreatments.** b** Group mean percent change in body weight from treatment initiation on Day 10 through end of study on Day 34. Error bars =  ± SEM. Significance reported for Day 34 versus untreated/vehicle controls; *ns* not significant, *p < 0.05, **p < 0.01, ****p < 0.0001

In order to evaluate the anti-tumor and anti-metastatic activity of SPD and anti-mCTLA-4 therapies alone and in combination, we treated groups of *n* = 10 4T1 tumor-bearing mice with IT vehicle, IT SPD (50 mg/kg), IP anti-mCTLA-4 (10 mg/kg), and IT SPD (50 mg/kg) + IP anti-mCTLA-4 (10 mg/kg), a group of untreated tumor-bearing mice were also included (Fig. 1a).

Treatment began on Day 10 at which point group mean tumor volumes ranged from 93 to 98 mm^3^. Animals were followed for 24 days after treatment initiation and the per animal change in TV was determined based on TV difference between Day 34 and Day 10. For each group, a mean change in TV (∆TV) was calculated based on individual animal data. The combination regimen had significantly reduced ∆TV (614 mm^3^; *p* < 0.0001) compared to vehicle control while SPD and anti-mCTLA-4 monotreatments resulted in ∆TV = 792 (*p* < 0.01 vs vehicle) and 970 mm^3^, respectively. The combination treatment ∆TV was also significantly reduced (*p* < 0.05) compared to untreated controls. Percent reductions in change in TV from treatment initiation to study end versus untreated and vehicle groups were 27% and 41% for SPD, 11% and 28% for anti-mCTLA-4, and 43% and 54% for combination treatment.

### Body weight, toxicity, and survival

Mean body weight change (Fig. 1b) between initiation of treatment and end of study for the vehicle control group was − 5% (median =  − 4%) and for animals in the untreated group mean weight change was − 3% (median = 0%). In comparison, animals who received the combination regimen showed a significant increase in body weight, gaining, on average, 11% (median = 10%; *p* < 0.01 and *p* < 0.05 vs. vehicle and untreated controls, respectively) by end of study. Monotherapies demonstrated smaller increase in mean body weight of 1% (median =  − 1.1%) and 2% (median = 3%) for SPD and anti-mCTLA-4, respectively.

Assessments of local toxicity and clinical observations of animal well-being were noted for each animal throughout study. Local effects of the orthotopic tumor with incidences of mild to moderate scabbing were found in 90–100% of animals in all groups. Except for animals receiving combination treatment, clinical characteristics of animal distress including rough pelage, piloerection, hunched posture, hind limb paralysis, and > 10% body weight loss were noted in all groups. Notably, administration of the combination regimen had no impact on animal well-being and was well tolerated as shown by significant animal weight gain as well as lack of adverse clinical findings.

Aside from one animal in the untreated group that was found dead on Day 34, all other animals survived to study end.

### Metastatic burden

**Fig. 2:**
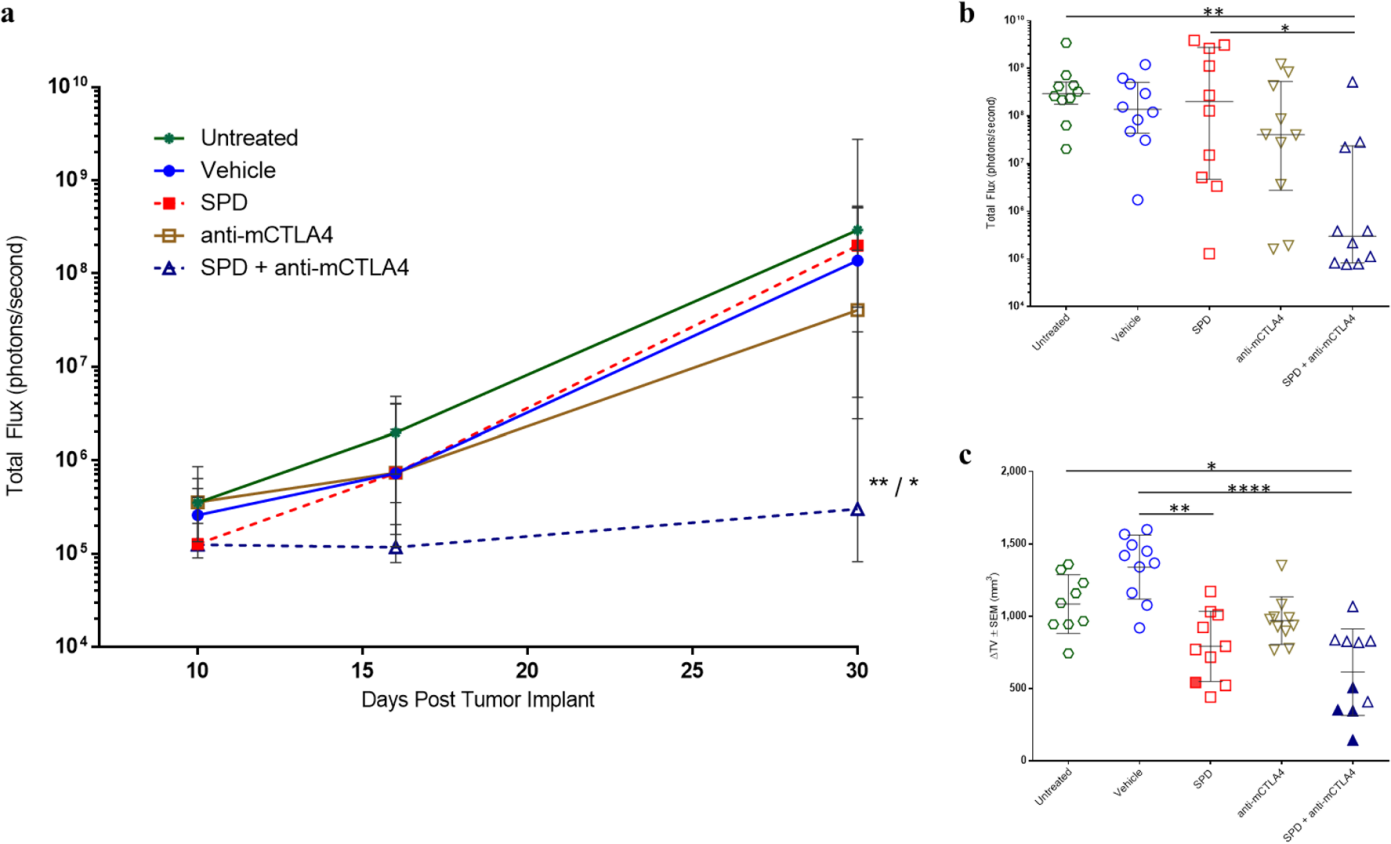
**a** Group median BLI ± IQR at Days 10, 16, and 30. *n* = 10 mice/group. Significance reported versus untreated controls/SPD; *p < 0.05, **p < 0.01. **b** Per animal Day 30 BLI with median and ± IQR shown. Significance reported as *p < 0.05, **p < 0.01. **c** Per animal ∆TV with group mean and SEM shown. Solid symbols indicate individual animals which did not have evidence of thoracic metastasis at Day 30. Significance reported as *p < 0.05, **p < 0.01, ****p < 0.0001

Metastatic spread to the thoracic cavity was monitored on Days 10, 16, and 30 using in vivo BLI (Fig. 2a). The SPD + anti-mCTLA-4 combination had significantly reduced Day 30 BLI compared to untreated controls (*p* < 0.01) and SPD (*p* < 0.05) groups. In this study, BLI signal above background (1.5E + 05 p/s) is indicative of metastatic spread to the thoracic cavity. On Day 30, in untreated, vehicle, and anti-mCTLA-4 groups, 100% of animals had evidence of metastasis as determined by BLI signal. One animal treated locally with IT SPD did not demonstrate thoracic metastasis. Remarkably, the combination of IT SPD + IP anti-mCTLA-4 therapies resulted in 40% of animals with no evidence of metastasis. A strong positive relationship was observed between reduction in tumor growth and reduction in metastatic spread, with animals that did not have evidence of thoracic metastatic spread and also having low ∆TV (Fig. 2b, c).

During necropsy, macroscopic evaluation of untreated animals found presumptive masses or nodules in the lungs, spinal column, and abdominal cavity in 80%, 30%, and 20% of animals, respectively. In addition, low (10%) incidences of metastasis were noted in tissue from the ovary, kidney, and thoracic cavity. IT vehicle-treated animals had macroscopically observed metastasis in the lungs (60%), spleen (30%), heart (20%), and thoracic cavity (20%), as well as one animal with a bilateral kidney mass. Similar extents of metastasis were found in SPD-treated animals in the lung (90%), spleen (50%), heart (20%), and thoracic cavity (20%), as well as low incidence of findings in kidneys, adrenal gland, and limbs. In the group treated with anti-mCTLA-4, metastasis was observed in the lungs (60%), spleen (20%), and thoracic cavity (20%). Two animals in this group were observed to have masses on the uterine horn (20%) and low incidences of metastasis were noted in the limbs and abdominal cavity. The combination regimen reduced lung metastasis with nodules noted in three of ten animals (30%) with low incidences of metastasis in the spleen (20%) and thoracic and abdominal cavities (10% each). These findings suggest that the combination therapy was effective at reducing incidence and extent of 4T1 metastasis following treatment initiation ten days after tumor implant.

### Flow cytometry—adaptive immunity

#### T cells

**Fig. 3:**
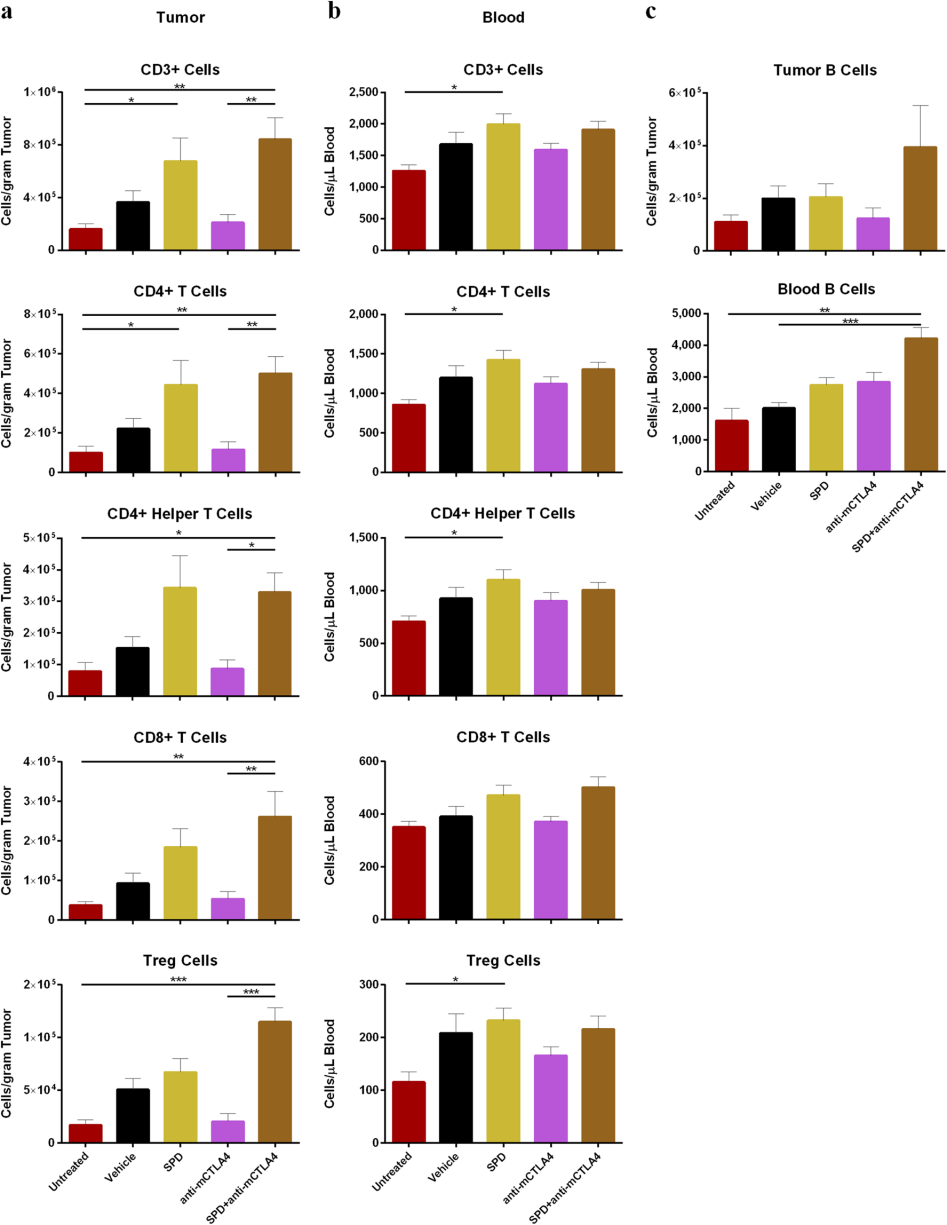
**a** Day 34 absolute counts of T cells in tumor-site tissue (untreated group *n* = 9 and mean TV = 1179 mm^3^ and all other groups *n* = 10 and group mean TV range = 712 mm^3^ (SPD + anti-mCTLA-4) to 1433 mm^3^ (vehicle)); mean + SEM. **b** Day 34 absolute counts of T cells in blood (*n* = 6 for untreated group, *n* = 9 for anti-mCTLA-4, and *n* = 10 for all other groups); mean + SEM. **c** Day 34 absolute counts of B cells in tumor-site tissue (top) and blood (bottom); mean + SEM. Significance reported as *p < 0.05, **p < 0.01, ***p < 0.001

The combination of IT SPD and systemic anti-mCTLA-4 mobilized T cells to the TME. In animals treated with the combination, all categories of CD3 + T cells evaluated were significantly increased (*p* < 0.05) compared to untreated controls and anti-mCTLA-4 alone (Fig. 3a). Densities of CD3 + T cells in the combination group were approximately 8.4 × 10^5^ cells/gram of tissue or 10% of CD45 + cells, and CD4 + T cells densities reached 5.0 × 10^5^ cells/gram of tissue or 6% of CD45 + cells (Fig. 3a, Online Resource Figure S1a). Absolute counts of tumor-associated CD4 + helper T cells were significantly increased in the combination treatment group of SPD + anti-mCTLA-4 (3.3 × 10^5^ cells/gram of tissue) versus anti-mCTLA-4 treatment alone (8.7 × 10^4^ cells/gram of tissue; *p* < 0.05) and untreated controls (7.9 × 10^4^ cells/gram of tissue; *p* < 0.05) (Fig. 3a). Treatment with SPD alone significantly increased absolute counts of tumor-associated CD3 + and CD4 + cells compared to untreated controls (*p* < 0.05) (Fig. 3a).

Combination treatment was also effective at recruiting CD8 + cytotoxic T cells, converting the TME from immunogenically “cold” to “hot.” The density of CD8 + T cells in the TME was fivefold higher in the combination group (2.6 × 10^5^ cells/gram of tissue) compared to anti-mCTLA-4 alone (5.3 × 10^4^ cells/gram of tissue; *p* < 0.01) and sevenfold increased versus untreated controls (3.7 × 10^4^ cells/gram of tissue; *p* < 0.01) (Fig. 3a). Following combination treatment, CD8 + T cells comprise 3% of all CD45 + cells and are significantly increased compared to anti-mCTLA-4 alone (*p* < 0.05) and untreated controls (*p* < 0.01) (Online Resource Figure S1a). Within the tumor-associated CD8 + T cell population, anti-mCTLA-4 treatment resulted in 73% CD69 + positivity which was significantly greater than SPD alone (43%; *p* < 0.01). No significant differences in tumor-associated CD8 + T cell PD-1 positivity or Ki67 + CD8 + T cells were detected between groups (Online Resource Figure S2a).

Local IT SPD treatment significantly increased the density of adaptive immune cells in blood compared to untreated controls (Fig. 3b). Mean absolute counts of peripheral CD3 +, CD4 +, and CD4 + helper T cells in SPD-treated animals were 1996, 1423, and 1102 cells/µL of blood, respectively, and were all significantly greater compared to untreated controls (*p* < 0.05) (Fig. 3b). The combination of SPD + anti-mCTLA-4 also increased the proportion of circulating T cells (Online Resource Figure S1b). CD3 + and CD4 + cells were significantly increased compared to anti-mCTLA-4 (*p* < 0.05) and untreated controls *(p* < 0.01). SPD alone resulted in increased populations of circulating CD3 + , CD4 + , and CD4 + helper T cells compared to untreated controls (*p* < 0.05).

The combination of SPD + anti-mCTLA-4 significantly increased the proportion of circulating CD8 + cytotoxic T cells compared to untreated and vehicle controls (*p* < 0.05) and the anti-mCTLA-4 monotreatment (*p* < 0.01) (Online Resource Figure S1b). Increase in circulating Ki67 +-proliferating CD8 + T cells were found in the groups treated with vehicle, SPD (*p* < 0.05 for both comparisons), and combination treatment (*p* < 0.01) compared to untreated controls. No significant differences in circulating CD8 + T cell CD69 or PD-1 positivity were detected between groups (Online Resource Figure S2b).

Within the tumor, the highest absolute counts of Tregs and proportion of total CD45 + cells were found in the combination treatment group (Fig. 3a, Online Resource Figure S1a), which was significantly greater compared to untreated controls (*p* < 0.001) and anti-mCTLA-4 (*p* < 0.001). Similar increases in Tregs were observed in the blood in groups treated with SPD ± anti-mCTLA-4 (Fig. 3b, Online Resource Figure S1b). In the tumor, CD8 + /Treg was significantly greater in anti-mCTLA-4 compared to vehicle treatment (*p* < 0.05) (Online Resource Figure S1c).

#### B cells

Although no significant differences in absolute counts or proportion of B cells was found within the tumor, the combination treatment trended toward an increase in tumor-associated B cells. Circulating B cells were most abundant in animals that received the combination regimen, which had an average of 4210 cells/µL of blood and were significantly increased compared to untreated controls (1609 cells/µL of blood; *p* < 0.01) and vehicle (2010 cells/µL of blood; *p* < 0.001) (Fig. 3c, Online Resource Figure S3).

### Flow cytometry—innate immunity

#### NK and NKT cells

**Fig. 4:**
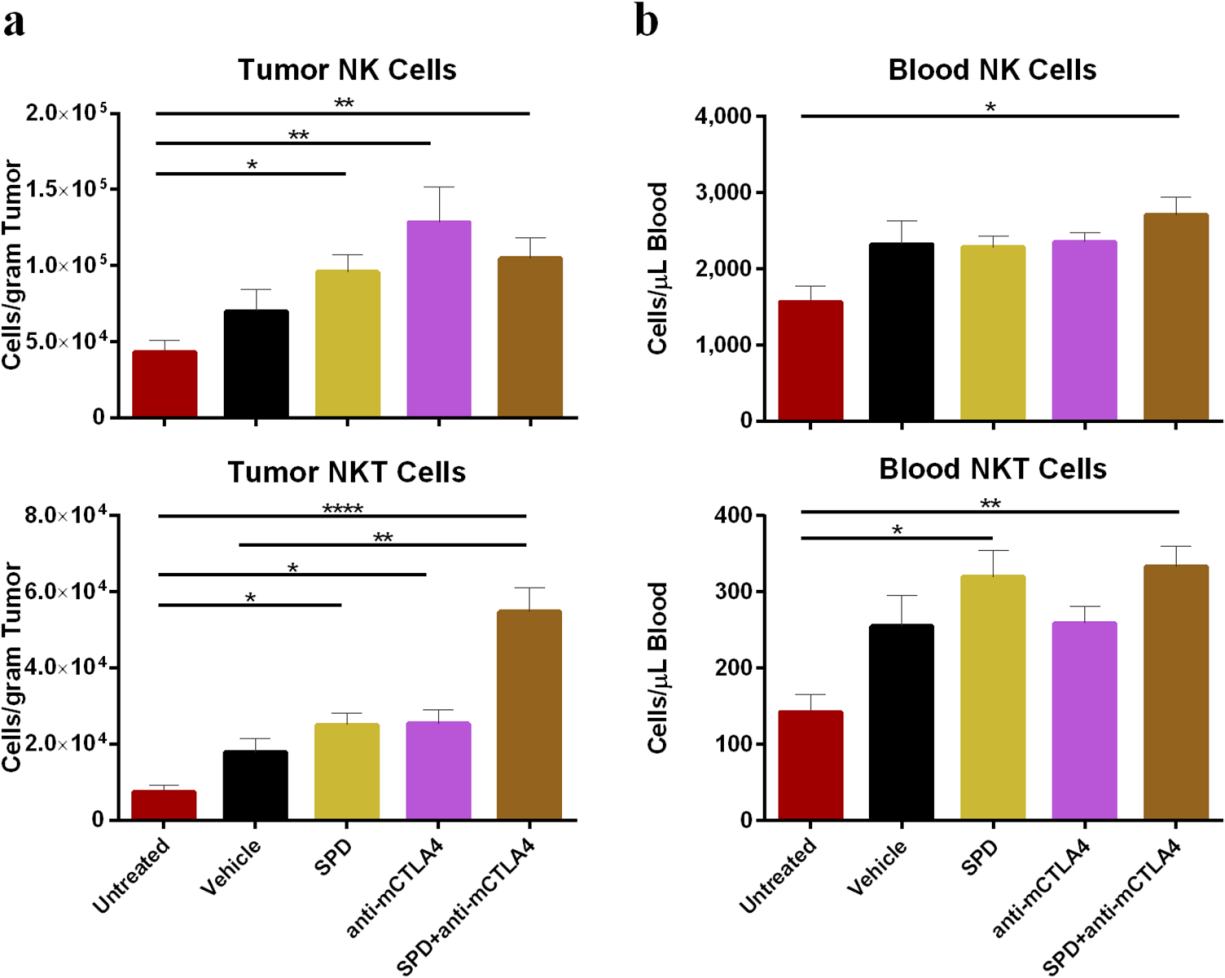
**a** Day 34 absolute counts of NK (top) and NKT (bottom) cells in tumor-site tissue; mean + SEM. For untreated group *n* = 9 and mean TV = 1179 mm^3^ and for all other groups *n* = 10 and group mean TV range = 712 mm^3^ (SPD + anti-mCTLA-4) to 1433 mm^3^ (vehicle). **b** Day 34 absolute counts of NK (top) and NKT (bottom) cells in blood; mean + SEM. *n* = 6 for untreated group, *n* = 9 for anti-mCTLA-4, and *n* = 10 for all other groups. Significance reported as *p < 0.05, **p < 0.01, ****p < 0.0001

Combination treatment resulted in increased density of NK and NKT cells in both the TME and circulating blood compared to untreated controls. In the TME, NKT cell densities were twofold higher in the combination group compared to either SPD or anti-mCTLA-4 groups and threefold (*p* < 0.01) or sevenfold (*p* < 0.0001) greater compared to vehicle and untreated controls, respectively (Fig. 4a). NK cell densities in the blood were greatest in the combination group (2704 cells/µL of blood) and were significantly increased compared to untreated controls (1564 cells/µL of blood; *p* < 0.05) (Fig. 4b). Circulating NKT cells were greatest in the groups treated with SPD ± anti-mCTLA-4 (333 and 320 cells/µL of blood) and were both significantly greater compared to untreated controls (142 cells/µL of blood; *p* < 0.05 for SPD alone and *p* < 0.01 for combination treatment) (Fig. 4b). The proportion of CD45 + cells in the tumor and blood identified as NKT cells were also greatest in the combination treatment group (0.71% and 0.18%, respectively) and were significantly increased compared to untreated (*p* < 0.001) and vehicle controls (*p* < 0.05) and in the case of blood also significantly greater compared to the anti-mCTLA-4-treated group (*p* < 0.05) (Online Resource Figure S4a-b). Percent of NK cells were greatest in the blood from the combination treatment (1.5%) and were significantly increased compared to untreated (*p* < 0.001) and vehicle controls (*p* < 0.05). SPD monotreatment had the second greatest proportion of NK cells in the blood (1.1%) and was significantly greater compared to untreated controls (*p* < 0.01) (Online Resource Figure S4 a-b).

#### Macrophages

**Fig. 5:**
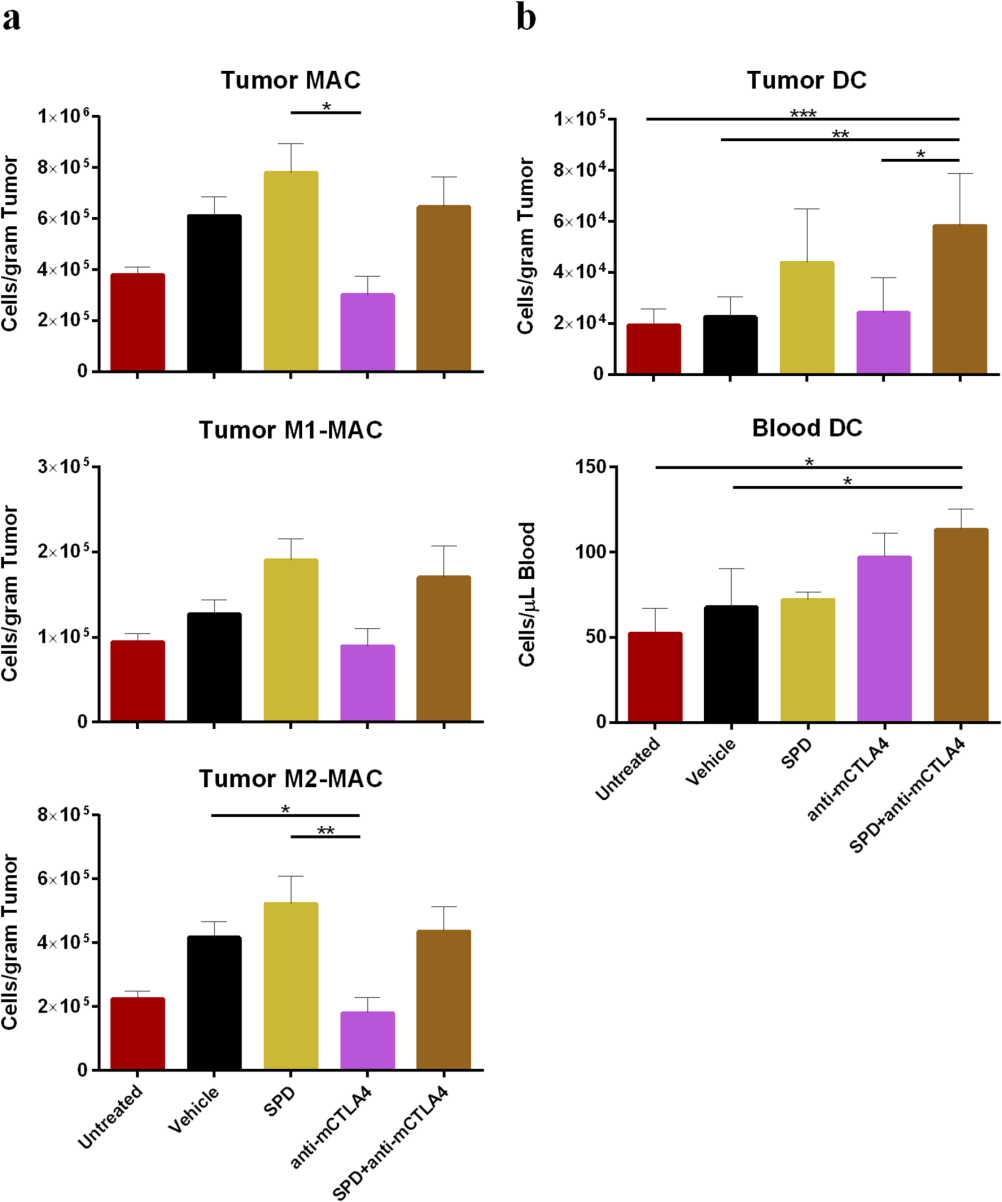
**a** Day 34 absolute counts of MAC (top), M1-MAC (middle), and M2-MAC (bottom) in tumor-site tissue; mean + SEM. For untreated group *n* = 9 and mean TV = 1179 mm^3^ and for all other groups *n* = 10 and group mean TV range = 712 mm^3^ (SPD + anti-mCTLA-4) to 1433 mm^3^ (vehicle). **b** Day 34 absolute counts of DC in tumor-site tissue (top; untreated group n = 9 and mean TV = 1179 mm^3^ and for all other groups *n* = 10 and group mean TV range = 712 mm^3^ (SPD + anti-mCTLA-4) to 1433 mm^3^ (vehicle)) and Day 34 absolute counts of DC in blood (bottom; *n* = 6 for untreated group, *n* = 9 for anti-mCTLA-4, and *n* = 10 for all other groups); mean + SEM. Significance reported as *p < 0.05, **p < 0.01, ***p < 0.001

Few significant differences in densities of tumor associated or circulating macrophages (MAC) were seen across treatment groups. IT SPD resulted in the greatest density in tumor-associated macrophages (TAM) at 7.8 × 10^5^ cells/gram tumor and was significantly increased compared to anti-mCTLA-4 monotreatment (3.0 × 10^5^ cells/gram tumor; *p* < 0.05). M2-MAC in SPD treatments were significantly increased compared to anti-mCTLA-4 (*p* < 0.01) which was reduced compared to vehicle control (*p* < 0.05) (Fig. 5a). Similarly, the SPD treatment resulted in the highest proportion of CD45 + tumor-associated MAC which were significantly increased *(p* < 0.05) compared to vehicle and anti-mCTLA-4. Anti-mCTLA-4 had the highest ratio of tumor-associated M1 to M2 MAC (0.58) which was significantly increased compared to vehicle control (*p* < 0.01). No significant differences were found in the levels of circulating macrophages. (Online Resource Figure S5a–c).

#### Dendritic cells

Combination of IT SPD and anti-mCTLA-4 resulted in the highest density of tumor associated and circulating DC (Fig. 5b). In the tumor tissue, DC were found at 5.8 × 10^4^ cells/gram tumor or 0.75% of total CD45 + cells for the combination treatment which were significantly increased compared to untreated (1.9 × 10^4^ cells/gram tumor or 0.37%, *p* < 0.01), vehicle (2.3 × 10^4^ cells/gram tumor or 0.34%, *p* < 0.01), and anti-mCTLA-4 (2.4 × 10^4^ cells/gram tumor or 0.43%, *p* < 0.05) (Online Resource Figure S6). In the blood, DC were most abundant in the combination group (113 cells/µL blood or 0.06% of total CD45 + cells) and were significantly increased compared to untreated and vehicle controls (*p* < 0.05 for absolute counts and %CD45 +, respectively) (Fig. 5b, Online Resource Figure S6).

#### Myeloid-derived suppressor cells

**Fig. 6:**
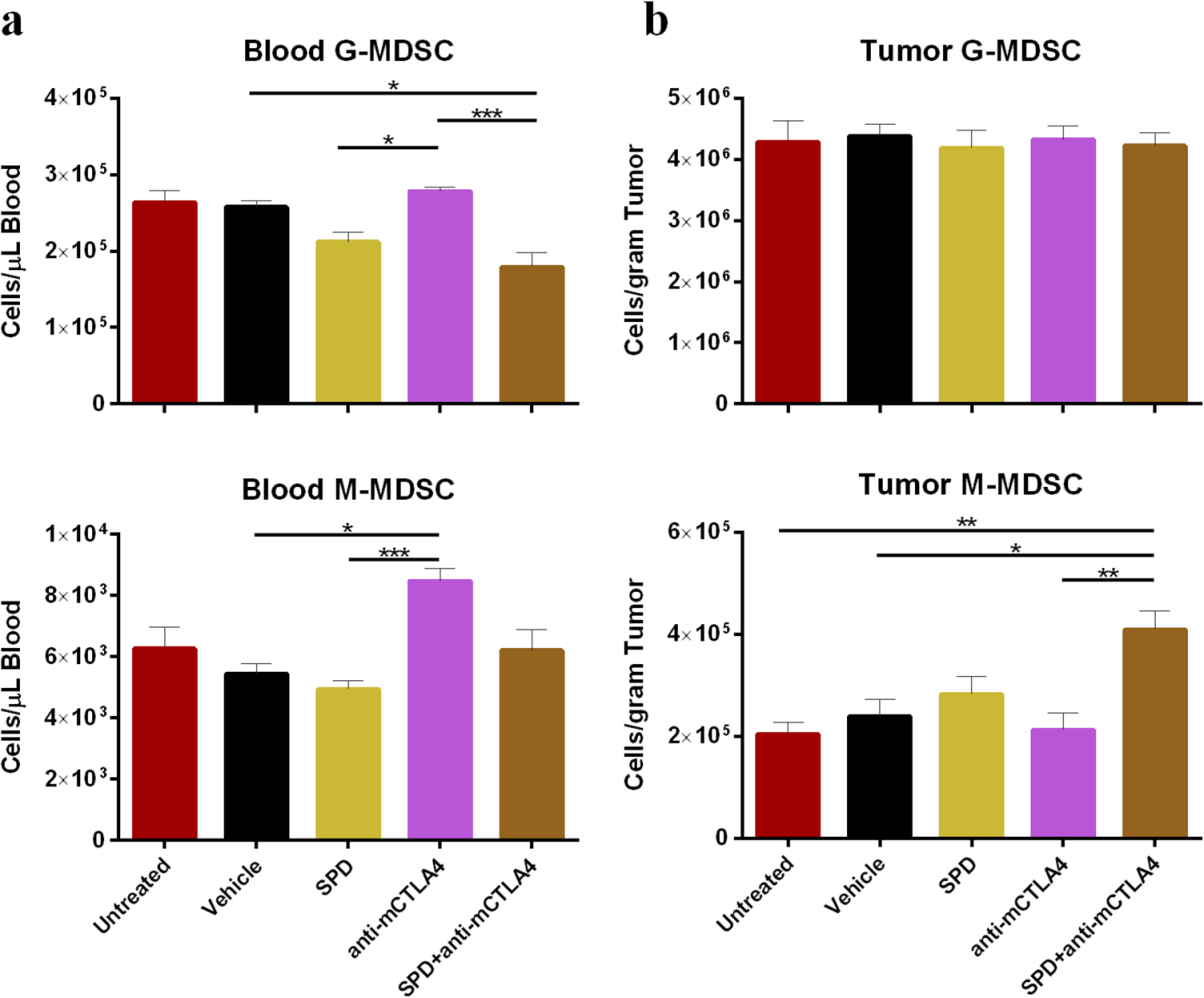
**a** Day 34 MDSC absolute counts and in blood (*n* = 6 for untreated group, *n* = 9 for anti-mCTLA-4, and *n* = 10 for all other groups); mean + SEM. **b** Day 34 absolute counts of MDSC in tumor-site tissue (untreated group *n* = 9 and mean TV = 1179 mm^3^ and for all other groups *n* = 10 and group mean TV range = 712 mm^3^ (SPD + anti-mCTLA-4) to 1433 mm^3^ (vehicle)); mean + SEM. Significance reported as *p < 0.05, **p <  0.01, ***p < 0.001

In the 4T1 model, combination of IT SPD and anti-mCTLA-4 significantly reduced absolute counts of immunosuppressive circulating MDSC (Fig. 6a). In the blood, G-MDSC (CD45 + CD19-CD11b + Ly6G +) were lowest in the combination group at 1.8 × 10^5^ cells/µL blood which was significantly reduced compared to anti-mCTLA-4-treated animals (2.8 × 10^5^ cells/µL blood; *p* < 0.001) and vehicle control (2.6 × 10^5^ cells/µL blood; *p* < 0.05). SPD monotreatment had 2.1 × 10^5^ G-MDSC cells/µL blood which was significantly reduced compared to anti-mCTLA-4 (*p* < 0.05) (Fig. 6a). Circulating M-MDSC (CD45 + CD19-CD11b + Ly6C +) were lowest in the SPD group (4.9 × 10^5^ cells/µL blood) and were significantly reduced compared to anti-mCTLA-4 (*p* < 0.001). Circulating M-MDSC were also significantly greater in anti-mCTLA-4 compared to vehicle treatment (*p* < 0.05) (Fig. 6a).

Intratumorally, there were no significant differences in G-MDSC density while M-MDSC were lowest in the untreated group (2.0 × 10^5^ cells/gram tumor) and significantly decreased compared to the combination treatment (4.1 × 10^5^ cells/gram tumor; *p* < 0.01) (Fig. 6b). However, as a proportion of total CD45 + population, G-MDSC were most decreased in the tumors and blood following combination treatment and were significantly reduced compared to untreated (*p* < 0.05), vehicle controls (bloods only; *p* < 0.01), and anti-mCTLA-4 (tumor only; *p* < 0.01) (Online Resource Figure S7a-b). In the blood, the proportion of CD45 + cells identified as M-MDSC were most reduced in the vehicle control group (1.8%) which was significantly lower compared to anti-mCTLA-4 (2.8%; *p* < 0.01) and combination treatment (3.2%; *p* < 0.0001), and SPD treatment alone was significantly reduced (*p* < 0.05) compared to combination treatment (Online Resource Figure S7b).

## Discussion

Reducing systemic exposure to chemotherapy while enhancing local tumoricidal effects remains a high priority in cancer therapy where regimens of IV chemotherapy are often limited by systemic toxicities. It has been postulated that local, sustained tumor treatment would make drug available to tumor cells over multiple cell-division cycles, resulting in greater tumoricidal benefits without compromising patient quality of life [16, 17]. However, success of many local chemotherapy regimens is challenged by limited drug delivery into tumor sites as well as abbreviated drug residence due, in part, to leakage of drug from the tumor and clearance by the immune system [5, 18]. Our goal is to prolong tumor residence time of taxanes by local administration of submicron particles of pure drug that act as a depot for continuous molecular drug release in the TME with gradual clearance from the tumor site at subtoxic levels.

In this study, SPD IT injections into orthotopic murine breast cancer implants in female BALB/c mice significantly reduced TV when given alone or in combination with anti-mCTLA-4. SPD treatments were associated with reduced orthotopic 4T1 TV compared to vehicle control with the greatest reduction in TV achieved with combination SPD + anti-mCTLA-4 treatment. The data suggest that abscopal effects may have occurred following combination treatment where extent of thoracic metastasis as measured by in vivo BLI was significantly reduced. Forty percent of the animals in the combination group had no evidence of lung metastasis and those same animals had the greatest reduction in primary TV at Day 34. As shown by significant animal weight gain and lack of adverse clinical findings, administration of the combination regimen was well tolerated.

The TME is a dynamic environment [19, 20]. Changes in the interactions between tumor, immune, and stromal cells occur as the size and structure of the tumor change over time. Death of cancer cells can be accompanied by changes in the composition of the cell surface and increased availability of soluble tumor-specific antigens [21]. Study results of IV immunotherapy used to treat solid carcinomas suggests that therapy targeting both the primary tumor and malignant cells could be effective in eliciting innate and long-lasting adaptive immunity resulting in disease regression [22–27]. Results of this study showed that, following SPD treatment, immune effector cells from both the innate and adaptive immune systems were increased in both the tumor and peripheral blood. The increase in primary tumor kill as well as reduction in metastasis with the addition of systemic anti-mCTLA-4 therapy suggests that disruption of the TME by SPD in combination with systemic immunotherapy produces a more effective anti-tumor response. The findings of this study, in addition to previous studies of SPD treatment in uro-oncologic xenografts [1], Renca xenografts [6] and SPP IT injections in pharmacology and clinical studies [5] support the hypothesis that submicron taxane particles are directly tumoricidal in the primary tumor and contribute to the innate and adaptive immune system’s therapeutic response by turning the primary “cold” tumor “hot” without creating local or systemic toxicity.

The relative increase in concentrations of CD4 + T cells and CD4 + helper T cells as well as CD8 + T cells in the tumor and peripheral blood following SPD (Fig. 3) is consistent with previous reports of increased concentrations of T cells in SPD-treated Renca xenografts [6]. These immune cell responses include increased density of CD4 + helper T cells, CD8 + T cells, B cells, NKT cells, and DC in the TME. Although SPD treatment increased T cell concentrations in the tumor accompanied by more modest increases in peripheral blood, the addition of anti-mCTLA-4 to IT SPD therapy did not result in an additional increase in T cell concentrations achieved in response to SPD treatment alone. The 4T1 orthotopic implant treated locally with submicron particle taxanes followed by systemic checkpoint inhibitors may be a useful model to study (1) T cell activation and exhaustion due to the continuous exposure of tumor cells to taxanes; (2) effect of persistent tumor antigen availability on constitution of the TME; (3) change in TME heterogeneity with varying dose schedules of SPD; and (4) comparison of immunotherapy effects as a function of time following changes in the TME including vascular and lymphatic access following SPD administration. These findings support the hypothesis that IT injections of long-acting taxane particles provide continuous tumoricidal levels which enhance the adaptive immune system’s response to availability of tumor-associated antigens from continuous and prolonged tumor cell kill.

Cytotoxic T lymphocytes and NK cells represent the major cytotoxic cell subsets involved in tumor cell death. NKTs and NK cells have the capacity to identify and destroy cancer cells in response to a wide variety of tumor-related stimuli [28–33]. In this study, following combination therapy, NKT cell concentrations increased in the tumor site and peripheral blood (Fig. 4). The significant decrease in both TV and BLI measures following combination therapy may have been due, in part, to local release of tumoricidal cytokines by the large numbers of NKT cells [30–32]. NK cell concentrations were shown to increase in both the tumor site with SPD or anti-mCTLA-4 monotreatment (Fig. 4). The concurrent increases in NK cell concentrations are consistent with reports that NK cells have been shown to arrive early in the TME and, together with DC, can facilitate an effective T cell tumoricidal response [29, 34, 35]. Notably, NK cells as effectors in treatment of solid tumors are typically limited by an inability to accumulate in the tumor [36]. It is possible that the tumoricidal contributions of NK, cytotoxic T cells, and NKT cells in the immune response to many forms of breast cancer [37–40] would be enhanced by IT administration of SPD into the primary tumor followed by systemic administration of an immunotherapy such as a checkpoint inhibitor.

In a study of adoptive T cell transfer therapy (ACT), Hu and Liu et al. [41] found that docetaxel given IV two days before ACT inhibited the T cell suppressive activity of MDSCs. Reductions in the blood concentrations of both G-MDSCs and M-MDSCs following SPD were also observed in this study (Fig. 6) which confirm and extend a previous report of MDSC reduction following SPD injection of Renca tumors [6]. Taken together, these results suggest that SPD injection into carcinomas may be an effective therapeutic approach to reduce MDSC-mediated immunosuppression.

In clinical evaluations, IV administration of docetaxel to patients with metastatic prostate or breast cancer improved outcomes and appeared to increase the ratio between T cells and Tregs, as well as reduce the immunosuppressive activity of Tregs in most patients, suggesting that selective use of immunotherapy in combination with specific chemotherapies, like docetaxel, may achieve a better clinical outcome than either alone [42–44]. Furthermore, IT injection of chemotherapy may be effective in treating solid tumors that are resistant to immunotherapy, especially if chemotherapeutic, like the submicron particle taxanes, is retained in the tumor releasing therapeutic concentrations of taxanes for weeks during tumor cell division [5, 6].

The data presented in this study, in which SPD IT injections into murine breast cancer tumors reduced TV and in combination with anti-mCTLA-4 further reduced primary tumor growth and the extent of thoracic metastasis, are consistent with the hypothesis that a necroptotic response of tumor cells to SPD may allow an influx of immune effector cells into the TME, perhaps in response to increased availability of tumor antigens [45]. Further, as these cells become available, they may be effective in treating metastatic disease, potentially providing a path forward for the development of effective combinations of therapies for solid tumors without added local and systemic toxicity.

## Supplementary Information

Below is the link to the electronic supplementary material.

Supplementary file1 (DOCX 450 kb)

## Data Availability

The datasets generated during and/or analyzed during the current study are available from the corresponding author on reasonable request.
