# Supplementary material for: Submicron particle docetaxel intratumoral injection in combination with anti-mCTLA-4 into 4T1-Luc orthotopic implants reduces primary tumor and metastatic pulmonary lesions
Source: Med Oncol. 2021 Jul 31;38(9):106. doi: 10.1007/s12032-021-01555-1 (PMC8325653; doi:10.1007/s12032-021-01555-1)
Supplement: Supplementary file 1 — Supplementary file1 (DOCX 450 kb) [file 12032_2021_1555_MOESM1_ESM.docx]

## **Supplementary Information (SI)**

Article Title: Submicron Particle Docetaxel Intratumoral Injection in Combination with anti-mCTLA-4 into 4T1-Luc Orthotopic Implants Reduces Primary Tumor and Metastatic Pulmonary Lesions

Journal Name: Medical Oncology

Author Names: Holly Maulhardt^1^ · Alyson Marin^1^ · Holly Hesseltine^1^ · Gere diZerega^1,2,*^

Affiliations:

^1^ US Biotest, Inc., 231 Bonetti Drive, Suite 240, San Luis Obispo, CA, USA

^2^ NanOlogy, LLC., 3909 Hulen Street, Fort Worth, TX, USA

^*^ Correspondence: gere.dizerega@usbiotest.com; Tel.: +01-805-595-1300

##



## **Fig. S1** **a** Day 34 % of CD45+ T cells in tumor-site tissue; mean + SEM. For untreated group n = 9 and mean TV = 1179 mm^3^ and for all other groups n = 10 and group mean TV range = 712 mm^3^ (SPD + anti-mCTLA-4) to 1433 mm^3^ (vehicle) **b** Day 34 % of CD45+ T cells in blood; mean + SEM. n = 6 for untreated group, n = 9 for anti-mCTLA-4 and n = 10 for all other groups. **c** Ratio of % CD45+ CD8+ T to Treg cells in tumor and blood. Significance reported as * = *p<0.05*, ** = *p<0.01*, *** = *p<0.001*, **** = *p<0.0001*





## **Fig. S2** **a** Day 34 % of CD8+ T cells with CD69+ in tumor-site tissue (top); % of PD1+ CD8+ T cells in tumor-site tissue (middle); median fluorescence intensity (MFI) for Ki67+ CD8+ T Cells in tumor-site tissue; mean + SEM. For untreated group n = 9 and mean TV = 1179 mm^3^ and for all other groups n = 10 and group mean TV range = 712 mm^3^ (SPD + anti-mCTLA-4) to 1433 mm^3^ (vehicle) **b** Day 34 % of CD8+ T cells with CD69+ in blood (top); % of PD1+ CD8+ T cells blood (middle); MFI for Ki67+ CD8+ T Cells in blood; mean + SEM. n = 6 for untreated group, n = 9 for anti-mCTLA-4 and n = 10 for all other groups. Significance reported as * = *p<0.05*, ** = *p<0.01*





## **Fig. S3** Day 34 % of CD45+ B cells in tumor-site tissue (top; untreated group n = 9 and mean TV = 1179 mm^3^ and for all other groups n = 10 and group mean TV range = 712 mm^3^ (SPD + anti-mCTLA-4) to 1433 mm^3^ (vehicle)) and in blood (bottom; n = 6 for untreated group, n = 9 for anti-mCTLA-4 and n = 10 for all other groups); mean + SEM. Significance reported as * = *p<0.05*, *** = *p<0.001*





**Fig. S4 a** Day 34 % of CD45+ NKT (top) and NK (bottom) cells in tumor-site tissue (n = 9 for untreated group and mean TV = 1179 mm^3^ and for all other groups n = 10 and group mean TV range = 712 mm^3^ (SPD + anti-mCTLA-4) to 1433 mm^3^ (vehicle)); mean + SEM. **b** Day 34 % of CD45+ NKT (top) and NK (bottom) in blood (n = 6 for untreated group, n = 9 for anti-mCTLA-4 and n = 10 for all other groups); mean + SEM. Significance reported as * = *p<0.05*, ** = *p<0.01*, *** = *p<0.001*, **** = *p<0.0001*





**Fig. S5** **a** Day 34 % of CD45+ MAC, M1-MAC and M2-MAC cells in tumor-site tissue (n = 9 for untreated group and mean TV = 1179 mm^3^ and for all other groups n = 10 and group mean TV range = 712 mm3 (SPD + anti-mCTLA-4) to 1433 mm^3^ (vehicle)); mean + SEM. **b** % of CD45+ MAC, M1-MAC and M2-MAC cells in blood (n = 6 for untreated group, n = 9 for anti-mCTLA-4 and n = 10 for all other groups); mean + SEM. **c** Ratio of M1-MAC to M2 MAC in tumor-site tissues (top) and blood (bottom); mean + SEM. Significance reported as * = *p<0.05*, ** = *p<0.01*





**Fig. S6** Day 34 % CD45+ DC in tumor-site tissue (top; n = 9 for untreated group and mean TV = 1179 mm^3^ and for all other groups n = 10 and group mean TV range = 712 mm^3^ (SPD + anti-mCTLA-4) to 1433 mm^3^ (vehicle)) and % CD45+ DC in blood (bottom; n = 6 for untreated group, n = 9 for anti-mCTLA-4 and n = 10 for all other groups); mean + SEM. Significance reported as * = *p<0.05*, ** = *p<0.01*





## **Fig. S7** Day 34 % CD45+ MDSC in tumor-site tissue (n = 9 for untreated group and mean TV = 1179 mm^3^ and for all other groups n = 10 and group mean TV range = 712 mm^3^ (SPD + anti-mCTLA-4) to 1433 mm^3^ (vehicle)); mean + SEM. **b** Day 34 % CD45+ MDSC in blood (n = 6 for untreated group, n = 9 for anti-mCTLA-4 and n = 10 for all other groups); mean + SEM. Significance reported as * = *p<0.05*, ** = *p<0.01, **** = p<0.0001*
